# Supplementary material for: Neutrophil Extracellular Traps in Pediatric Inflammatory Bowel Disease: A Potential Role in Ulcerative Colitis
Source: Int J Mol Sci. 2024 Oct 16;25(20):11126. doi: 10.3390/ijms252011126 (PMC11507660; doi:10.3390/ijms252011126)
Supplement: Supplementary file 1 [file ijms-25-11126-s001.zip › ijms-3224124-supplementary.pdf]

Supplementary Material

MATERIALS AND METHODS

Immunofluorescent Staining using Histone 3 cleavage Antibody

Neutrophils were stained with immunofluorescence using a novel antibody targeting a NET-specific histone H3 cleavage (Tilley, D. O et al; 2022). Briefly, cells were permeabilized using PBS (pH 7.5) containing 0.5% v/v Triton X-100 for 3 minutes, followed by three 5-minute washes in PBS. The cells were then blocked with 10% blocking buffer (5% w/v BSA, 5% w/v human albumin, and 0.05% v/v Tween-20 in PBS) for 30 minutes at room temperature. Primary antibody staining was performed by incubating the samples overnight at 4°C with 3D9 mouse monoclonal antibody (1 µg/ml) diluted in 1% blocking buffer. Coverslips were washed (3x5minutes) with PBS before incubating with secondary antibody for 2 hours at room temperature in the dark (Invitrogen, a10037, 1:500 in blocking buffer). After a final wash in PBS, nuclei were counterstained with DAPI for 10 minutes at room temperature. Slides were washed for 5 minutes in PBS and mounted using Immuno-mounting medium for subsequent imaging.

SUPPLEMENTARY FIGURES

Supplementary Figure S1

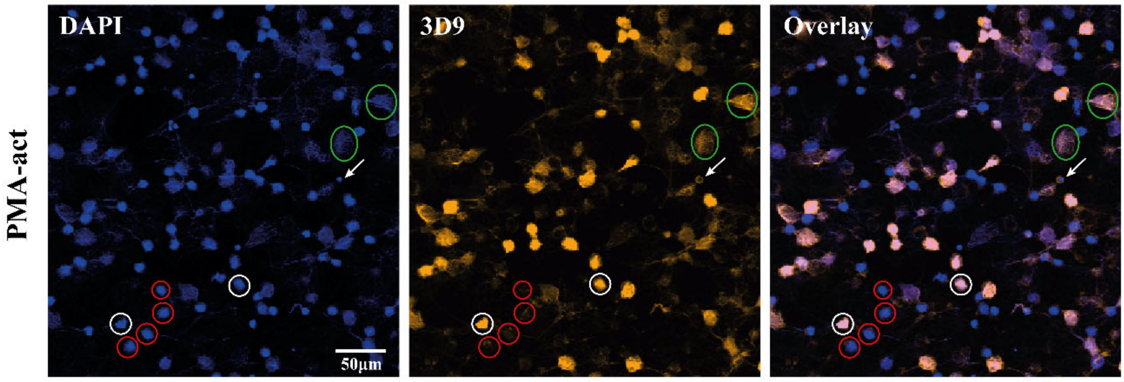

Figure S1. NET Formation by Neutrophils from Pediatric UC Patients, analysis using Histone 3 cleavage Antibody

Neutrophils isolated from patients with UC at diagnosis displayed high NET formation upon stimulation with PMA, with similar % of NETs forming neutrophils as shown in figures 4A and 4D (Sytox Green staining).

(not-netting neutrophil=red circle; early stage NETosis with compacted nucleus = white arrow; advanced NETosis with compacted nucleus=white circle; late-stage NETosis=green circles) (Scale bar 50 and 100 µm)
